# Text-Book of Ophthalmology

**Published:** 1899-08

**Authors:** 


TEXT-BOOK OF OPHTHALMOLOGY. By Dr. ERNEST FUCHS, Professor of Ophthalmology in the University of Vienna. Authorised translation, revised from the seventh enlarged and improved German edition, by A. DUANE, M. D., Assistant Surgeon Ophthalmic and Aural Institute, New York With 277 illustrations. 8vo, 860 pages. Second American edition. New York: D. Appleton & Co. 1899.

It is seven years since the first American edition of this text-book was presented to the profession, and during that time five German editions have been issued. This is ample proof, in itself, of the high esteem in which the work is held in Germany, and the demand for another American edition shows the favor with which it is received in this country. The present edition is a great improvement over the previous ones, both on account of the revisions made by the author and the additions made by the translator. Much new matter has thus been incorporated, and the contributions of the translator, notably those on heterophoria and correction of errors of refraction, will be especially acceptable to the American practitioner. As the work now stands, it is a quite full exposition of the anatomy and physiology of the eye, and of its pathology and medical and surgical therapeutics. What has been disregarded by the author, has been supplemented, in great measure, by the translator in his discriminating notes, and in this way both the German and American ideas are set forth.

The text-book as here presented is a scholarly work, and merits a foremost position among the numerous and excellent treatises on diseases of the eye. No one can study it without great profit, and no one having anything to do with the treatment of ocular diseases can afford to be without it. Not only is it a work valuable for what it contains, but it is also clearly translated, and is finely illustrated and printed. A. A. H.

